# Effects of exercise-based prehabilitation in children undergoing elective surgeries: a systematic review

**DOI:** 10.12688/f1000research.74493.2

**Published:** 2022-01-10

**Authors:** Jean Noronha, Stephen Samuel, Vijay Pratap Singh, H Shivananda Prabhu

**Affiliations:** 1 Department of Physiotherapy, Kasturba Medical College, Mangalore, Manipal Academy of Higher Education, Manipal, India; 2 Department of Physiotherapy, Kasturba Medical College, Mangalore,, Manipal Academy of Higher Education, Manipal, India; 3 Department of Physiotherapy, Kasturba Medical College Mangalore, Manipal Academy of Higher Education, Manipal, India; 4 Department of Surgery, Kasturba Medical College, Mangalore, Manipal Academy of Higher Education, Manipal, India

**Keywords:** Pre-operative exercises, preoperative exercise, prehabilitation, exercise therapy, surgery, paediatric, children, adolescents

## Abstract

**Background:** Prehabilitation is a therapeutic strategy involving preoperative physical exercises, nutritional support, and stress and anxiety reduction. This approach has been gaining popularity and has been seeing effective results in adults in terms of improving pre and postoperative outcomes. The purpose of this review was to summarise the evidence about the effects of exercise-based prehabilitation programs on various outcome measures in children post elective surgeries.­­

**Methods**: PubMed, Scopus, Web of Science, PEDro, CINAHL/EBSCO and EMBASE electronic databases were searched from inception to June 2021. Based on the inclusion criteria, titles and abstracts were independently screened by the authors. After that, a data extraction table of the selected studies which included the participants, type, and details of exercise intervention, outcome measures and results were analysed after which the quality assessment of the studies was done.

**Results: **The search yielded 2219 articles of which three articles fulfilled the inclusion criteria with two studies being randomized controlled trials and one being a quasi-experimental pre-post type of study. One randomized controlled trial was on the effects of exercise-based prehabilitation in reducing pulmonary complications post cardiac surgeries in children and the other two studies were on the effects of prehabilitation on functional capacity & pulmonary function. All the three articles found that exercise-based prehabilitation had a positive effect on children’s post-surgery.

**Conclusion: **Although there is a paucity of evidence-based literature, we conclude based on the existing literature retrieved by our review that exercise-based prehabilitation improves postoperative outcomes and helps in reducing postoperative complications in children undergoing various surgeries.

## Abbreviations used

ACBT: Active Cycle of Breathing Technique

CG: Control Group

FEV1: Forced Expiratory Volume 1

FEV1/FVC Ratio: Tiffeneau-Pinelli index

FVC: Forced Vital Capacity

IG: Interventional group

PEFR: Peak expiratory flow rate

POP: Postoperative Physiotherapy

POPE: Preoperative Physiotherapy Education

PRISMA: Preferred Reporting Items for Systematic Reviews and Meta-Analyses

RCT: Randomized control trial

ROM: Range of Motion

TUGT: Time up and Go test

6MWT: Six-minute walk test

9SCT: 9 Step climbing test

10MWT: 10-minute walk test

## Introduction

Major surgeries in children along with the deleterious effects of the condition that predisposes a child for the surgery lead to complications that need to be therapeutically managed in children.

Prehabilitation is a multimodal type of approach that helps a patient planned for any major surgery and also allows them to prepare to, manage the stressors in the pre-surgical period and also undertake the necessary rehabilitation successfully so that they can return to their pre-operative state with better and improved outcomes. ^1^^,^^2^ Prehabilitation encompasses pre-operative physical exercises, nutritional support, and stress and anxiety reduction. ^3^^,^^4^ The concept of prehabilitation dates as far back as World War II and was initially started not as a part of the pre-surgical procedure. ^1^ Prehabilitation has its first mentions in articles in 1942 where it raised the fact that military recruits would be medically screened and treated with respect to their health and comorbidities, resulting in a higher number of acceptances. ^1^^,^^5^ and came to light post-2011 after the systematic review published by Valkenet *et al.* about prehabilitation before joint, cardiac and abdominal surgeries ^3^^,^^6^

Prehabilitation/preoperative exercise is a set of interventions done before surgery that helps the patient to be prepared for post-surgical stressors and also help improve their functional capacity (FC) through the exercises. ^1^^,^^7^ Patients’ ability to function to their fullest capacity can deteriorate because of inactivity during the surgical period and even if the surgery has been successful there can be chances of deconditioning. ^1^^,^^8^^,^^9^ Current studies and reviews done in prehabilitation concerning the adult population do show that there is improvement in the post-operative complications and length of stay and also in their post-operative pain. ^3^^,^^10^ Therefore the concept of prehabilitation is said to not only help the patient prepare themselves before a major surgery for post-surgical complications, but it also helps the patient to understand the importance of it to reduce the complications, helping them promote physical fitness and also optimize their psychological wellbeing. This also helps the patients return to their normal levels of functionality that were present before surgery. ^11^^–^^13^

This review aims at examining the current body of evidence in the area of exercise-based prehabilitation in children undergoing various elective surgeries.

## Methods

### Search strategy

A data search was made on PubMed, Scopus, Web of Science, PEDro, EMBASE, CINAHL/EBSCO from inception to June 2021. The terms used for search for the pediatric population were the following: infant [Mesh], child [Mesh], adolescent [Mesh], children. The terms preoperative exercise [Mesh], exercise [Mesh], exercise therapy [Mesh], breathing exercises [Mesh], preoperative exercises [Mesh] were used related to the intervention and for the population type: general surgery [Mesh], paediatrics/surgery [Mesh] and surgical procedures operative [Mesh] were the terms used. The search terms were combined with a Boolean operator ‘AND’ or ‘OR’ wherever applicable. The references of the included articles were also screened for possible relevant studies.

### Selection criteria

The articles were screened based on the following pre-set criteria. The inclusion criteria include 1) Studies that included participants in the age group of 0-18 years; 2) Studies that include children undergoing elective surgeries; 3) Studies published in English language and 4) were either randomized control trial (RCT), Non-RCT, single group post, case study and case series and the exclusion criteria included were 1) studies with participants undergoing a prehabilitation program other than exercise 2) studies that included participants above 18 years.

## Results

### Characteristics of studies

**Figure 1.**
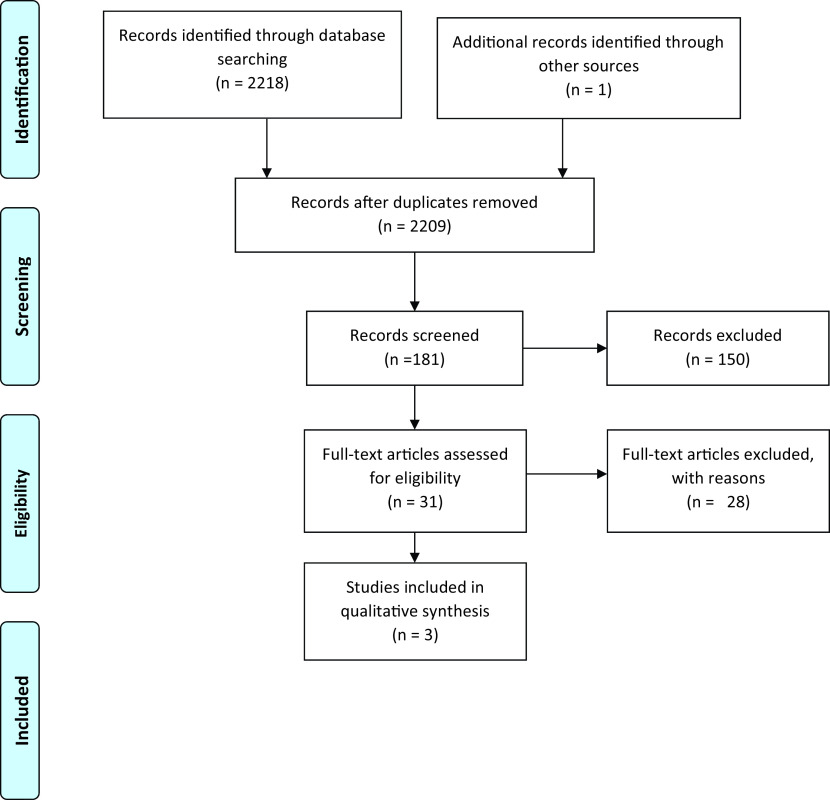
PRISMA flow chart.

All the data retrieved from the databases, summing up to 2219 articles, were fed in the Mendeley Desktop v1.19.8 after which duplicates were removed. The articles were then screened through the titles and 181 articles were found eligible, following this the abstract screening removed 150 articles, after which full-text screening was done, and 29 papers were excluded, eventually yielding three papers that meet the inclusion criteria of this review. The PRISMA flow chart as in Figure 1. Outlines details regarding the identification, screening, eligibility, and inclusion of the studies in this review.

### Qualitative analysis

**Figure 2.**
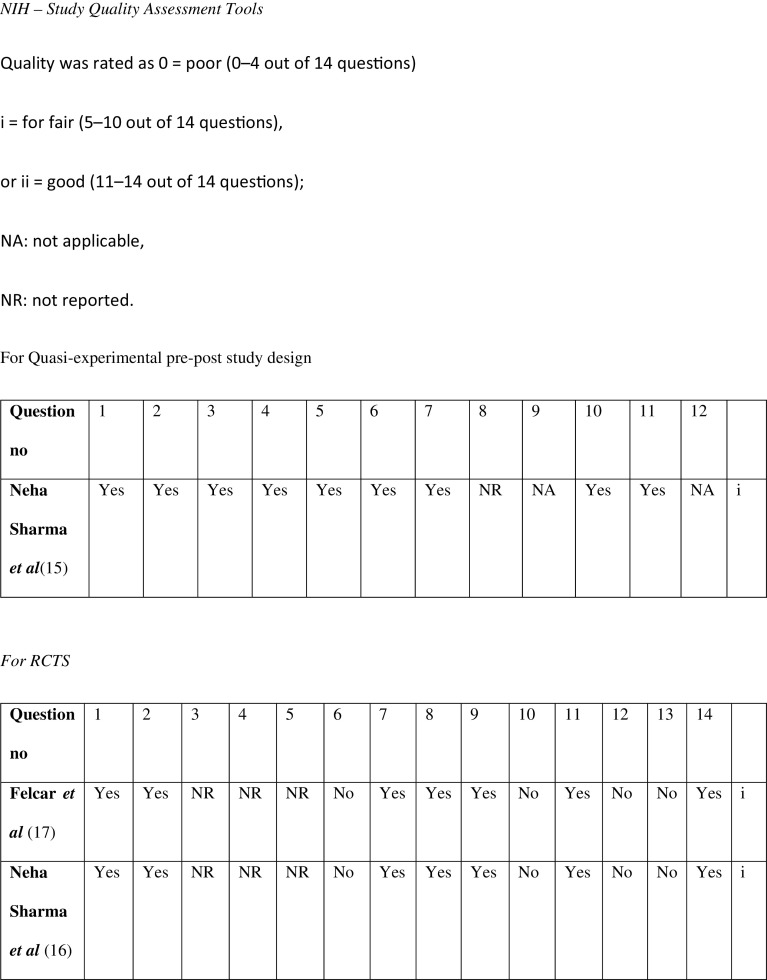
NIH study quality assessment tool. ^14^

The risk of bias scoring was done using the NIH Quality assessment scales as shown in Figure 2. ^14^ The quality assessment scales were used depending on the type of study. Two separate scales were used for the pre-post study design and the RCT. The scales covered everything regarding the type, duration of the study, the sample sizes, characteristics of the population and about its randomization, the interventions used, and whether participants and therapists were blinded. A score of 9/12 was rated for the pre-post type of study done by Sharma N *et al*. ^15^

A score of 7/14 was given for the RCT done by Sharma N *et al.* ^16^ and Felcar *et al.* ^17^respectively.

### Population of the included studies

Of the three studies, the two studies included children scheduled for abdominal surgeries in the age group 5-17 years of age ^15^^,^^16^ and the other study had children one-day-old to six-year-old with congenital heart disease who underwent heart surgeries. ^17^

### Intervention of the included studies

All of the three studies included exercise-based prehabilitation as the main form of intervention. Of the three studies, one study focused on the use of exercise-based prehabilitation in reducing pulmonary complications through chest physiotherapy, clearance techniques, support and guidance to parents, and early mobilization. ^17^ The other two studies included the N-PARP protocol ^15^ used for prehabilitation This protocol included exercises to be given from the pre-operative period till POD5 and included breathing exercises, ROM exercises, and ambulation. ^15^^,^^16^

### Outcomes of the included studies

Felcar *et al*. ^17^ included the presence or absence of pulmonary complications as its major outcome measure. while Sharma N *et al.* ^15^^,^^16^ had a pulmonary function and functional capacity (FC) as their main outcomes which included spirometer values and Six-minute walk test (6MWT) respectively and others being 10-minute walk test (10MWT), Timed up and go test (TUGT), chest expansion. A detailed explanation of the studies is given in Table 1.

**Table 1.** Summary of the exercise-based prehabilitation studies.

Author	Study type	Participants	Interventions	Exercise and dosages	Outcome measures		
Josiane Marques FELCAR, *et al.*	RCT	135 completed M = 73 F = 62 Congenital heart disease Age = 1 day old – 6 years	IG = Pre-operative and Postoperative physiotherapy	IG = *Pre-op-ex:* ‐ two sessions ‐ clearance & expansion techniques, abdominal support, and advice to parents + *post-op ex:* airway clearance, pulmonary re-expansion and early mobilization	*Primary*		
					Pulmonary Complications		
					Yes-17 (25%) No-51 (75%)		Yes-29 (43.3%) No-38 (56.7%)
					Pneumonia		
			CG = postoperative physiotherapy		7 (10.3%)		13 (19.4%)
					Atelectasis		
					6 (8.8%)		8 (11.9%)

					Both		
					4 (3.9%)		8 (11.9%)
					*Secondary*		
					Time on mechanical ventilation		
					35.8 (7-204)		36 (8-288)
					Time of stay in the ICU		
					6 days (3-13.7)		6 days (3-17)
					Total time of hospital stay		
					13.5 (7-27.7)		14 (8-44)
					Duration of surgery		
					142.5 (93.7,193.7)		140 (100,240)
					Time of CPB		
					41 (21.5-69.7)		41 (0-90)
					other complications (breathing -, sepsis and surgical site infection)		
					Yes-16 (23.5) No-52 (76.5)		Yes-35 (52.2) No-32 (47.8)
Neha Sharma, *et al*	One group pre-test–a post-test, quasi-experimental pilot trial	8 Participated M = 6 F = 2 All Open abdominal surgery Age = 5-17 years children	IG = POPE + POP	IG = POPE: Deep breathing, trunk, pelvic mobility and leg ROM exercises. ‐ 30min ‐ 1 session ‐ 1 day before surgery + POP: deep breathing &, segmental breathing exercises, ACBT, limb ROM, and early ambulation program	*IG*	*Pre*	*Post*
					*Primary*		
					Spirometry		
					FVC		
					83.8 (74.1–93.4)		82.3 (71.9–92.7)
					FEV1		
					83.0 (69.3–96.3)		80.1 (68.7–91.6)
					FEV1/FVC RATIO		
					105.1 (94.8–115.5)		100.4 (88.0–112.8)
					PEFR		
					68.2 (52.5–84.0)		67.7 (52.9–82.5)
					*Secondary*		
					6MWT		
					464 (438–490)		417 (389–445)
					Borg’s scale		
					6.4 (6.1–6.8)		5-7.2 (6.2–8.0)
					10MWT		
					12.9 (11.4–14.4)		15.9 (13.3–18.5)
					TUGT		
					12.5 (11.2–13.9)		18.7 (15.2–22.2)
					Chest expansion – T2		
					2.3 (1.9–2.8)		2.2 (1.8–2.6)
					T4		
					3.7 (3.0–4.5)		3.3 (2.4–4.2)
					T10		
					4.6 (4.0–5.2)		2.6 (2.1–3.2)
					9SCT		
					13.8 (10.0–17.6)		18.7 (14.0–23.4)

Author	Study type	Participants	Interventions	Exercise and dosages	Outcome measures			
Neha Sharma, *et al*.	RCT	21 included IG = 11 CG = 10 All Open abdominal surgery Age = 5-17 years children	IG: POPE + POP CG: POP	IG = POPE: Deep breathing, trunk, pelvic mobility and leg ROM exercises. + POP: deep breathing &, segmental breathing exercises, ACBT, limb ROM, and early ambulation program CG = POP: deep breathing &, segmental breathing exercises, ACBT, limb ROM, and early ambulation program	*IG*		*CG*	
					*Pre*	*Post*	*Pre*	*Post*
					*Primary*			
					Spirometry: FVC			
					74 (72.5, 91)	77 (68, 93.5)	62 (49.5, 106.0)	56 (48, 67)
					FEV1			
					87 (63, 103)	83 (60.5, 95.5	57 (37.0,65.0)	58 (48.5,65.5)
					FEV1/FVC RATIO			
					106 (89,117)	98 (82, 116)	92 (64.0, 106.0)	89 (66, 102.5)
					PEFR			
					75 (41, 96)	70 (44.5, 85.5)	32 (29.0,53.0)	46 (38.5,72.5)
					*Secondary*			
					6MWT			
					469 (409, 492)	410 (380, 413	423 (365, 513)	381 (341, 468)
					10mWT			
					12.7 (11.5, 14.3)	16.9 (14.6, 18.9)	13.1 (11.8,14.8)	19 (17.1,20.2
					TUGT			
					12.9 (11.4, 14.1)	20.8 (17.2, 23.9)	16.2 (11.7,19.8)	21.8 (18.7, 24.3)
					Chest expansion – T2			
					2.3 (2.0, 2.7)	2.1 (2.0, 3.0)	-2.0 (1.5, 2.1)	1.5 (1.5, 2.0
					T4			
					3.9 (3.1, 4.6)	3.8 (2.2, 4.4)	2.9 (1.7, 3.3)	2.3 (1.8, 2.7)
					T10			
					4.8 (4.2, 5.5)	4.0 (3.5, 4.9)	4.1 (3.1, 4.3)	3.0 (2.7, 3.6)
					9SCT			
					12.9 (10.5, 16.7)	17.4 (16.0, 25.7)	15.3 (13.9, 16.0)	18.8 (17.0, 21.6)

Abbreviations: RCT - Randomized control trial; IG - Interventional group; CG - Control Group; POPE - Preoperative Physiotherapy Education; POP - Postoperative Physiotherapy; ACBT - Active Cycle of Breathing Technique; ROM-Range of Motion; FVC-Forced Vital Capacity; FEV1 - Forced Expiratory Volume 1; FEV1/FVC Ratio - Tiffeneau-Pinelli index; PEFR - Peak expiratory flow rate; 6MWT - Six-minute walk test; 10MWT - 10-minute walk test; TUGT - Time up and Go test; 9SCT - 9 step climbing test.

### Data extraction

A data extraction table was made to summarize and cover all the details regarding the participants, study design, sample size, study groups, type and dosage of exercise intervention, outcomes measures, and conclusion for all the selected studies. A detailed description of the Data extraction is presented in Table 1.

## Discussion

This systematic review aimed at identifying studies that gave an exercise-based prehabilitation intervention to children undergoing various surgeries. While searching articles for this review various studies were found that included post-operative exercise after surgery in children, but very few studies included prehabilitation in the routine clinical care of these patients.

Sharma N *et al.* published two studies in 2020 ^15^ and 2021 ^16^ about the effects of prehabilitation on pulmonary function and FC in the patients undergoing elective abdominal surgeries and Felcar *et al.* studied its effects in the reduction of post-op pulmonary complications in children undergoing cardiac surgery. ^17^

In the two studies conducted by Sharma N *et al*. ^15^^,^^16^ the effects of prehabilitation on FC and pulmonary functions were studied using a spirometer as a measurement tool. There was a trend seen in both the studies that no major changes in the values of the spirometer (that includes Forced Vital Capacity (FVC), Forced Expiratory Volume 1 (FEV1), Tiffeneau-Pinelli index (FEV1/FVC ratio), Peak Expiratory Flow Rate (PEFR)) were seen in the pre-surgical period and on POD 5, but there was a decline seen from the pre-operative period to POD 1 and from POD1 to POD5. The only difference seen in the RCT ^16^ compared to the pre-post study ^15^ conducted by Sharma N *et al*. was that FVC improved post prehabilitation and surgery. Values of chest expansion seemed to be better in the IG than CG in the RCT. ^16^ Lastly, one of the common findings in both the studies was that the values of 10MWT, TUGT, 9SCT were seen better in the CG of both studies rather than in the IG. ^15^^,^^16^ This study implies that exercise-based prehabilitation, when given in a proper format and incorporated well in routine care can have beneficial effects in children during the post-operative period.

In the study done by Felcar *et al.*, ^17^ its was seen that children in the CG were seen to have a higher frequency of developing pulmonary complications such as pneumonia or atelectasis or both as compared to the IG that received both the treatment options, i.e., prehabilitation and post-operative exercises. ^17^ This implies that children who received exercise-based prehabilitation have a lesser frequency of developing any other complications as compared to the children that didn’t receive prehabilitation.

Quality assessment of each of the studies was done by using the NIH Quality assessment scale. Two different scales were used for each type of study i.e. the pre-post type of study and the other for an RCT ^14^ The Pre-post study was done by Neha *et al*. ^15^ had a scoring of 9/12 which acc to their scale was categorized as fair. This study included clearly stated objectives, had pre-set inclusion and exclusion criteria. The sample size was around 12 participants but enough to conclude about the effects of the N-PARP (prehabilitation protocol) in that set population and to conclude that the study could be done in a larger population. there is also no information regarding the blinding of the populations in this study.

The NIH Quality assessment is done for The articles of Felcar *et al*., ^17^ and Neha *et al.* ^16^ scored them (7/14) and (7/14) respectively which according to their scale belonged to the fair category. Both these RCTs did not have any details regarding the blinding and concealment of the participants.

## Conclusion

Although few in number, the available literature leads us to the conclusion that exercise-based prehabilitation plays an important role in improving health-related outcome measures in children undergoing various surgeries.

## Data availability

### Underlying data

All data underlying the results are available as part of the article and no additional source data are required.

### Reporting guidelines

Open Science Framework: PRISMA Checklist final.docx, https://doi.org/10.17605/OSF.IO/B3CPX.

Data are available under the terms of the Creative Commons Attribution 4.0 International license (CC-BY 4.0).
