# Supplementary material for: Effects of exercise-based prehabilitation in children undergoing elective surgeries: a systematic review
Source: F1000Res. 2022 Jan 10;10:1262. Originally published 2021 Dec 8. [Version 2] doi: 10.12688/f1000research.74493.2 (PMC8861468; doi:10.12688/f1000research.74493.2)
Supplement: Peer review history

# Peer review: Reviewer response for version 2

**DOI:** 10.5256/f1000research.120065.r122900

**Published:** 2022-02-21

**Authors:** Anna-Maria Platschek

**Affiliations:** 1 Institute of Movement and Neurosciences, German Sport University Cologne, Cologne, North Rhine-Westphalia, Germany

In my opinion, this is a brilliant systematic review aimed at examining the effects of exercise-based prehabilitation involving physical exercise, nutritional support, and stress and anxiety reduction, on various outcome measures in children undergoing elective surgeries.

 The authors introduce the topic on the basis of many literature references. In the definition of prehabilitation (introduction, 3rd paragraph), it should be added, if necessary, that the exercise interventions can be performed actively by the patient himself or by a therapist. Especially in children under 3 years of age (compare participants in study Felcar *et al.* 2008), the intervention is more likely to be performed by a (physio)therapist.

 All required steps of a systematic review were followed. It is a bit unclear why so many articles (especially full-text articles) were excluded. In Figure 1 it is only described that it happened "with reasons". It would be interesting to know if there were two or three categories/main reasons why the articles had to be excluded from the review.

 In the subsequent discussion, the results obtained are very well discussed in the light of the literature. In addition, it should be critically discussed that in the studies of Sharma N *et al.* 2020, 2021, only eight participants took part in the study.

 In summary, it is an excellent review with results not yet found in the existing literature.

Are the rationale for, and objectives of, the Systematic Review clearly stated?

Yes

Is the statistical analysis and its interpretation appropriate?

I cannot comment. A qualified statistician is required.

Are sufficient details of the methods and analysis provided to allow replication by others?

Yes

Are the conclusions drawn adequately supported by the results presented in the review?

Yes

Reviewer Expertise:

Exercise Science

I confirm that I have read this submission and believe that I have an appropriate level of expertise to confirm that it is of an acceptable scientific standard.

# Peer review: Reviewer response for version 1

**DOI:** 10.5256/f1000research.78256.r102366

**Published:** 2022-01-05

**Authors:** Forhad Akhtar Zaman

**Affiliations:** 1 Department of Community Medicine & Family Medicine, All India Institute of Medical Sciences (AIIMS), Guwahati, Assam, India

I think this is an excellent systematic review aimed at reviewing studies of exercise-based prehabilitation intervention to children undergoing various surgeries.

 The strength of the study was that the review was done very meticulously and it followed all the required steps of systematic review. This will be an important addition to the existing literature. As the selection criteria was very robust & specific, only three out of a whole lot of studies could be selected for the review. Although few in number, I really like the way in which the discussion was done and evidence presented. I hope the authors will correct the few numerical errors which which has been mentioned in the report.

 I have only a couple of minor points of clarification:

1. In the Methodology section, it was not mentioned why only randomized controlled trials & quasi-experimental studies were selected. 
2. There is a mismatch between the number of total eligible titles (181) & total removed (150), thus full-text articles assessed for eligibility should have been 31, but it is mentioned as 33 in the PRISMA Flow chart (Fig-1). Also, out of 33 full-text articles which was assessed for eligibility, 29 papers were excluded, leaving 4 papers that ultimately met the inclusion criteria, but it is mentioned as 3.

Are the rationale for, and objectives of, the Systematic Review clearly stated?

Yes

Is the statistical analysis and its interpretation appropriate?

I cannot comment. A qualified statistician is required.

Are sufficient details of the methods and analysis provided to allow replication by others?

Partly

Are the conclusions drawn adequately supported by the results presented in the review?

Yes

Reviewer Expertise:

Communicable disease especially TB, HIV, Covid

I confirm that I have read this submission and believe that I have an appropriate level of expertise to confirm that it is of an acceptable scientific standard.

# Response

**Published:** 2022-01-06

**Authors:** Stephen Samuel

**Affiliations:** Manipal Academy of Higher Education, India

Dear F1000Research Team,

 We thank the reviewer and the editorial team for taking out the time to conduct an in-depth review of our article. We thank you for the appreciation you have given us regarding this systematic review. We also thank you for the clarifications. The necessary changes have been made and addressed accordingly. The modified manuscript and the latest PRISMA flow chart with changes tracked in the word document have been uploaded.

Reviewer Comment 1

*"In the Methodology section, it was not mentioned why only randomized controlled trials & quasi-experimental studies were selected."*

**Reply –** The following types of study designs, i.e., RCT (Randomized Controlled Trials), Non-RCT, Single group Pre-post, Case study, and series were to be included; but during the data extraction and reviewing process only RCT and quasi-experimental studies were retrieved

Reviewer Comment 2

*"There is a mismatch between the number of total eligible titles (181) & total removed (150), thus full-text articles assessed for eligibility should have been 31, but it is mentioned as 33 in the PRISMA Flow chart (Fig-1). Also, out of 33 full-text articles which was assessed for eligibility, 29 papers were excluded, leaving 4 papers that ultimately met the inclusion criteria, but it is mentioned as 3."*

**Reply-** We thank the reviewer for the correction and we have made the necessary changes as suggested.
